# Differences in *Acinetobacter baumannii* Strains and Host Innate Immune Response Determine Morbidity and Mortality in Experimental Pneumonia

**DOI:** 10.1371/journal.pone.0030673

**Published:** 2012-02-08

**Authors:** Anna de Breij, Matthieu Eveillard, Lenie Dijkshoorn, Peterhans J. van den Broek, Peter H. Nibbering, Marie-Laure Joly-Guillou

**Affiliations:** 1 Department of Infectious Diseases, Leiden University Medical Center, Leiden, The Netherlands; 2 Study group for host pathogens interactions, Department of Bacteriology, Angers University Hospital, Angers Medical School, Angers University, Angers, France; Institut de Pharmacologie et de Biologie Structurale, France

## Abstract

Despite many reports documenting its epidemicity, little is known on the interaction of *Acinetobacter baumannii* with its host. To deepen our insight into this relationship, we studied persistence of and host response to different *A. baumannii* strains including representatives of the European (EU) clones I–III in a mouse pneumonia model. Neutropenic mice were inoculated intratracheally with five *A. baumannii* strains and an *A. junii* strain and at several days morbidity, mortality, bacterial counts, airway inflammation, and chemo- and cytokine production in lungs and blood were determined. *A. baumannii* RUH875 and RUH134 (EU clone I and II, respectively) and sporadic strain LUH8326 resulted in high morbidity/mortality, whereas *A. baumannii* LUH5875 (EU clone III, which is less widespread than clone I and II) caused less symptoms. *A. baumannii* type strain RUH3023^T^ and *A. junii* LUH5851 did not cause disease. All strains, except *A. baumannii* RUH3023^T^ and *A. junii* LUH5851, survived and multiplied in the lungs for several days. Morbidity and mortality were associated with the severity of lung pathology and a specific immune response characterized by low levels of anti-inflammatory (IL-10) and specific pro-inflammatory (IL-12p40 and IL-23) cytokines at the first day of infection. Altogether, a striking difference in behaviour among the *A. baumannii* strains was observed with the clone I and II strains being most virulent, whereas the *A. baumannii* type strain, which is frequently used in virulence studies appeared harmless.

## Introduction

Multidrug-resistant *Acinetobacter baumannii* is a cause of severe infections in critically ill patients and notorious for its ability to spread epidemically. Three clonal lineages of *A. baumannii*, European (EU) clone I, II and III, are implicated in outbreaks worldwide [1], [2]. Other *Acinetobacter* species, including the skin colonizer *A. junii* [3], are only incidentally involved in infection [4].

Various factors are assumed to contribute to the ability of *A. baumannii* to colonize the hospital environment and patients [5]–[11]. However, knowledge on the host's response to *A. baumannii* is limited. Recognition by Toll-like receptor 4 and CD14 [12] and early recruitment of neutrophils [13], [14] are important factors in the host innate defence against respiratory *A. baumannii* infection in mice. Others demonstrated the differential ability of clinical *A. baumannii* isolates to induce severe infections in neutropenic mice [15]. We previously showed *in vitro* that *A. baumannii* strains induce significantly less inflammatory cytokine production in human airway epithelial cells and cultured human macrophages than *A. junii* strains do [5], emphasizing the role of the innate immune system in *A. baumannii* infections.

The aim of the present study was to investigate the virulence of and host innate immune response to well-characterized *A. baumannii* strains, including representatives of clones I–III, and an *A. junii* strain in a mouse pneumonia model.

## Results

### Morbidity and mortality

Mice infected with the different *A. baumannii* strains and the *A. junii* strain (Table 1) were monitored daily for morbidity and mortality. Mice infected with *A. baumannii* RUH3023^T^ or *A. junii* LUH5851 showed virtually no signs of morbidity (Table 2). In contrast, infection with *A. baumannii* clone I (RUH875) and clone II (RUH134) caused high morbidity already at the first day of infection, which remained high during the second day. Infection with clone III (LUH5875) and sporadic isolate LUH8326 was accompanied by significantly (p<0.01) less morbidity at the first day of infection. For LUH8326, morbidity increased during the second day of infection but for LUH5875 it remained low (Table 2).

**Table 1:** Strain characteristics.

Strain	City (country)		Year	Specimen	EU Clone*	Outbreak†	MDR‡
***A. baumannii***							
**UH875**	Dordrecht	(NL)	1984	urine	I	+	+
**RUH134**	Rotterdam	(NL)	1982	urine	II	+	+
**LUH5875**	Utrecht	(NL)	1997	blood	III	+	+
**LUH8326**	Leiden	(NL)	2002	wound	-	−	−
**RUH3023^T^ (ATCC19606^T^)**	Atlanta	(USA)	1965	urine	-	−	−
***A. junii***							
**LUH5851**	Leiden	(NL)	1999	ear	-	−	−

*Strain belonging to European clones I–III (+) or not belonging to these clones (−). All isolates have been identified to species by one or more genotypic methods [1], [33], [34].

† Outbreak-associated (+) strain (i.e., common AFLP profile in >2 patients and with same time-space-origin) or (−) sporadic strain.

‡ Multidrug-resistant (+) strain (i.e., resistant to more than two of the following drug classes or combinations: cephalosporins, carbapenems, ampicillin-sulbactam, quinolones and aminoglycosides) or (−) susceptible strain.

**Table 2:** Morbidity and lung pathology associated with *Acinetobacter* respiratory infection.

	*A. baumannii*					*A. junii*
	RUH875 (clone I)	RUH134 (clone II)	LUH5875 (clone III)	LUH8326 (sporadic)	RUH3023^T^	LUH5851
**Clinical score** *						
Day 1	2.7±1.0	2.9±0.9	1.9±1.2	2.3±0.9	0.0±0.2	0.0±0.2
Day 2	3.2±0.6	2.7±1.2	2.2±1.2	3.3±0.5	0.0	0.1±0.2
Day 3	NA	NA	1.9±1.3	1.5±1.7	0.0	0.0
Day 4	NA	NA	0.2±0.4	NA	0.0	0.0
**Lung pathology score** †						
Day 1	8.2±0.7	8.0±0.9	6.8±0.5	7.2±0.0	2.1±0.9	3.6±1.1
Day 2	8.0±0.9	8.3±0.8	6.9±1.5	7.1±0.3	2.1±0.6	3.0±1.2
Day 3	NA	NA	7.3±0.6	7.5±1.2	2.2±0.2	2.7±0.6
Day 4	NA	NA	6.7±1.0	NA	2.4±0.4	2.5±0.5

*Morbidity was recorded daily using a clinical score, which includes mobility, the development of conjunctivitis, and aspects of the hair and ranges from 0 for no clinical symptoms to 4 for maximal symptoms.

† Sections of lungs of mice were stained with haematoxylin & eosin and lung tissue damage was assessed using the lung pathology score, which includes alveolar wall destruction, leukocyte infiltration and hemorrhage and ranges from 0 for no pathology to 9 for severe pathology.

Values are means ± standard deviations for 8 mice except for LUH8326 at day 3, where n = 4. Values are representative for surviving mice only. NA, not assessable, due to the high mortality associated with these strains.

**Figure 1:**
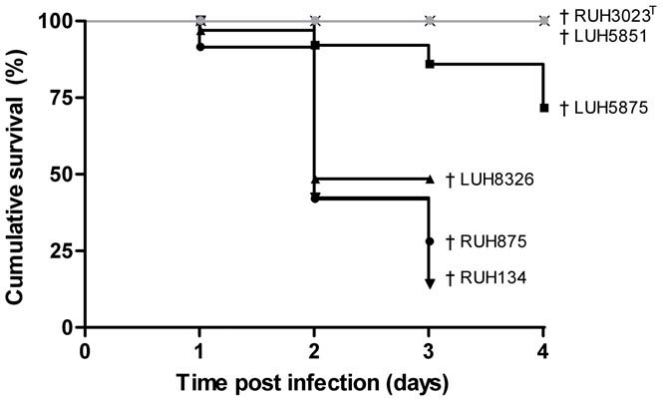
Survival associated with *Acinetobacter* respiratory infection. Survival of mice after intratracheal infection with *A. baumannii* clone I strain RUH875, clone II strain RUH134, clone III strain LUH5875, sporadic strain LUH8326 and type strain RUH3023^T^, and *A. junii* strain LUH5851. Results are expressed as percentage survival at days 1–4 of infection. †, all mice dead/sacrificed.

Mice infected with RUH3023^T^ or *A. junii* LUH5851 did not die, whereas mortality was very high among mice infected with LUH8326, RUH875, and RUH134 (52%, 72% and 86%, respectively, Fig. 1). Less animals (28%) died after infection with LUH5875 (p<0.05, Fig. 1). Most animals died within the first 2 days of infection with RUH875, RUH134 and LUH8326 and between days 3–4 of infection with LUH5875. Of note, the results described in the next paragraphs are representative for the surviving mice only.

### Persistence of Acinetobacter strains in lungs and extrapulmonary dissemination

**Figure 2:**
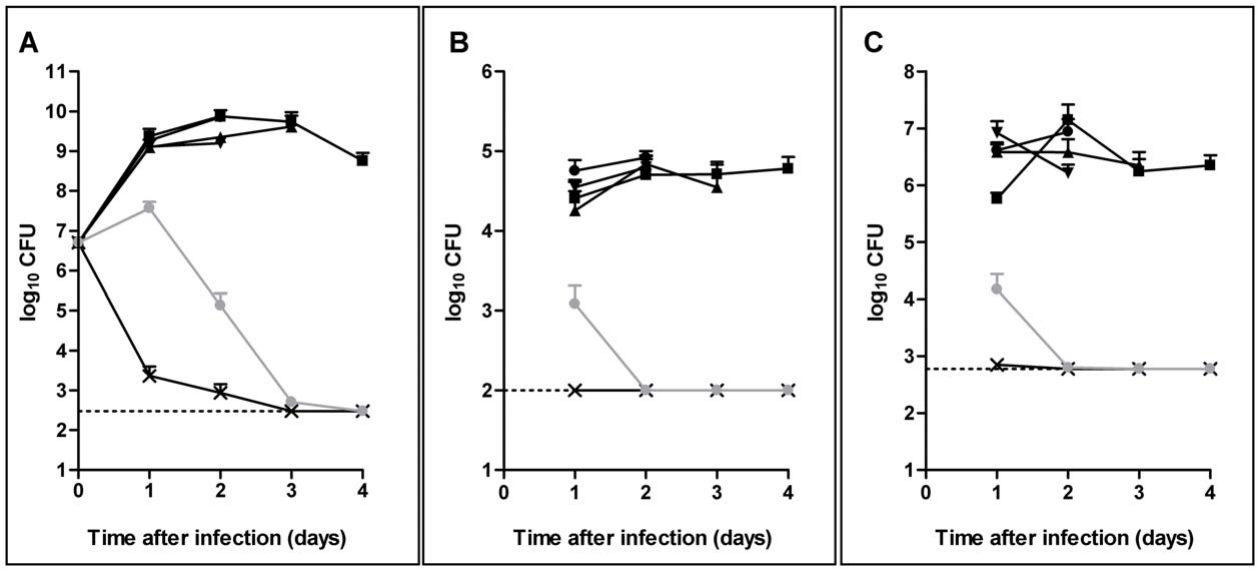
Persistence of *Acinetobacter* strains in the lungs and extrapulmonary dissemination. Levels of *A. baumannii* clone I strain RUH875 (black circles), clone II strain RUH134 (black downward triangles), clone III strain LUH5875 (black squares), sporadic strain LUH8326 (black upward triangles) and type strain RUH3023^T^ (black crosses), and *A. junii* strain LUH5851 (gray circles) in the lungs (A), bloodstream (B) and spleen (C) of mice at 1 to 4 days after intratracheal injection. Results are expressed as mean CFU per g of tissue (for lung and spleen) or CFU per ml (for blood) ± standard errors of the mean of 8 mice except for LUH8326 at 3 days after infection, where n = 4. Values are representative for surviving mice only. Dotted lines represent the lowest limit of detection.

After 24 h of infection, RUH875, RUH134, LUH5875 and LUH8326 had multiplied in the lungs to approximately 1×10^9^ CFU/g of lung (Fig. 2A). These strains persisted in the lungs up to day 3–4 of infection (Fig. 2A). In contrast, RUH3023^T^ was cleared from the lungs already within the first day of infection. The levels of *A. junii* LUH5851 remained stable during the first day of infection, declined sharply thereafter with complete clearance by day 3.

RUH875, RUH134, LUH5875, LUH8326 and *A. junii* LUH5851 were found in blood and spleen after the first day of infection (Fig. 2B, C). The bacterial load in the spleen after the first day of infection was significantly (p<0.05) higher for RUH875 than for LUH5875. *A. junii* LUH5851 disseminated into the blood and spleen to a significantly (p<0.05) lower extent than RUH875, RUH134, LUH5875 and LUH8326. For these *A. baumannii* strains, levels in blood and spleen remained stable up to day 3–4, whereas *A. junii* LUH5851 was cleared completely from blood and spleen already at day 2 of infection. *A. baumannii* RUH3023^T^ did not disseminate into the blood and spleen. Overall, bacterial counts in lungs correlated (p<0.01) to those in the bloodstream (r = 0.85) and spleen (r = 0.85). Furthermore, morbidity and mortality were associated (p<0.01) with bacterial counts in the lungs (r = 0.77 and r = 0.62, respectively), bloodstream (r = 0.86 and r = 0.75, respectively) and spleen (r = 0.85 and r = 0.82, respectively).

### Lung pathology

**Figure 3:**
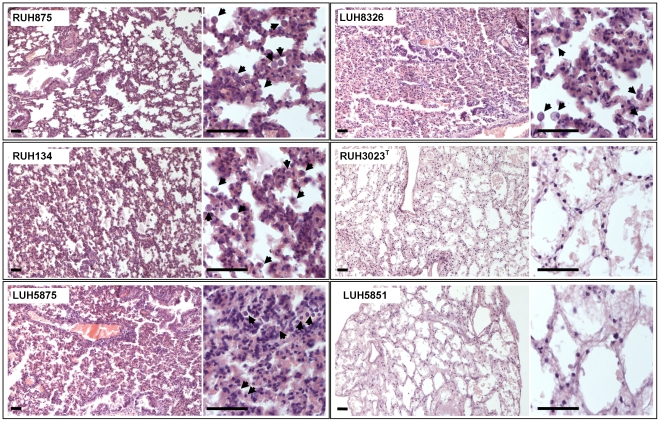
Light micrographs of lungs of mice infected with *Acinetobacter*. Sections of the lungs of mice at 1 day after infection with *A. baumannii* clone I strain RUH875, clone II strains RUH134, clone III strain LUH5875, sporadic strain LUH8326, type strain RUH3023^T^ or *A. junii* strain LUH5851, stained with haematoxylin-eosin. Arrows indicate inflammatory cell infiltrates. Bars, 50 µm.

Histologic examination of the lungs of mice at the first day of infection with RUH875, RUH134, LUH5875 and LUH8326 revealed hypercellularity due to increased numbers of lymphocytes, monocytes and macrophages, and thickened alveolar walls (Fig. 3, Table 2). The lungs of mice infected with these strains were highly consolidated and many areas had hemorrhagic lesions. The severity of tissue damage remained stable over time for all strains (Table 2). Alveolar wall destruction was more severe after the first day of infection with RUH875 and RUH134 than with LUH5875 and after the second day of infection with RUH875 and RUH134 than with LUH8326. Infection with RUH3023^T^ and *A. junii* LUH5851 resulted in significantly (p<0.05) less lung damage (Table 2).

Overall, severity of tissue damage was correlated (p<0.001) to bacterial levels in the lungs (r = 0.81), morbidity (r = 0.81) and mortality (r = 0.84).

### Inflammatory response to Acinetobacter

After the first day of infection with *A. baumannii* strains RUH875, RUH134, LUH5875 and LUH8326, levels of all chemo- and cytokines in the lungs were substantially elevated (p<0.05) as compared to basal levels. The levels of the chemokines KC, MIP-1α, MIP-2, and the pro-inflammatory cytokines IL-1β, IL-6 and TNFα further increased and peaked at day 2 of infection (Table 3). The kinetics of production of the anti-inflammatory IL-10 differed significantly in animals infected with the four strains with a peak at day 1 in animals infected with LUH8326, at day 2 after infection with RUH134 and at day 3 after infection with LUH5875. At the first day of infection, levels of IL-10 in lungs were significantly (p<0.05) lower in mice infected with RUH875 and RUH134 as compared to infection with LUH5875 and LUH8326. IL-10 levels remained lower after the second day of infection with RUH875 and RUH134 as compared to LUH5875. Moreover, levels of the anti-inflammatory IL-13 in lungs of mice infected for 1 day with RUH875 tended to be lower (p = 0.1) than with LUH5875. In addition to these anti-inflammatory cytokines, lower (p<0.05) levels of the pro-inflammatory IL-12p40 and IL-23 were seen in lungs of mice infected for 1 day with RUH875 and RUH134 than with LUH5875 and LUH8326. *A. baumannii* RUH3023^T^ and *A. junii* LUH5851 did not induce cyto- and chemokine production (data not shown).

**Table 3:** Inflammatory response in lungs of mice infected with four virulent *A. baumannii* strains.

		RUH875 (clone I)		RUH134 (clone II)		LUH5875 (clone III)		LUH8326 (sporadic)	
Chemokines	Day								
**KC** †	**1**	386	(38–700)*	311	(198–474)*	377	(4–555)*	310	(28–493)*
basal: 17 (3–57)	**2**	326	(150–749)*	236	(16–306)*	186	(25–540)*	204	(7–1753)*
	**3**	NA		NA		52	(8–224)	45	(32–422)
	**4**	NA		NA		41	(4–45)	NA	
**MIP-1α** †	**1**	142	(1–404)	114	(13–287)*	112	(13–217)*	147	(6–201)*
basal: 2 (1–5)	**2**	589	(118–888)*	319	(5–1076)*	760	(3–909)*	196	(2–765)*
	**3**	NA		NA		78	(12–654)*	124	(90–596)*
	**4**	NA		NA		114	(5–191)*	NA	
**MIP-2** †	**1**	171	(4–246)*	145	(71–372)*	119	(3–257)*	137	(6–220)*
basal: 5 (1–45)	**2**	400	(200–755)*	342	(4–591)*	365	(6–546)*	312	(3–406)*
	**3**	NA		NA		79	(4–253)*	109	(86–219)*
	**4**	NA		NA		97	(3–202)*	NA	
**RANTES** ††	**1**	1753	(975–5282)*	1682	(893–2708)*	2124	(1406–2452)*	1885	(996–3340)*
basal: 236 (221–501)	**2**	1229	(794–1760)*	1278	(671–1773)*	1559	(1475–1627)*	1000	(812–1381)*
	**3**	NA		NA		930	(691–1010)*	958	(665–2027)*
	**4**	NA		NA		834	(646–850)*	NA	
**Pro-inflammatory cytokines**									
**IL-1β** †	**1**	20	(5–60)*	19	(7–66)*	18	(5–31)*	30	(5–48)*
basal: 1 (1–2)	**2**	51	(13–264)*	33	(3–135)*	69	(6–268)*	24	(2–81)*
	**3**	NA		NA		19	(3–84)*	28	(12–75)*
	**4**	NA		NA		14	(4–20)*	NA	
**IL-6** †	**1**	133	(3–217)*	67	(19–280)*	85	(2–181)*	119	(1–174)*
basal: 3 (2–11)	**2**	127	(43–670)*	76	(4–395)*	98	(0.4–376)*	72	(1–151)*
	**3**	NA		NA		10	(1–217)	6	(3–266)
	**4**	NA		NA		9	(1–14)	NA	
**IL-12p40** ††	**1**	1901	(1202–3091)*	2249	(743–4130)*	2824	(1818–5251)*	3698	(1900–5365)*
basal: 203 (163–237)	**2**	1730	(377–2380)*	1513	(976–2644)*	1991	(1093–3005)*	1031	(834–1282)*
	**3**	NA		NA		669	(381–1853)*	760	(415–2210)*
	**4**	NA		NA		319	(116–494)	NA	
**IL-23** †	**1**	99	(58–113)*	82	(48–122)*	150	(104–175)*	113	(98–150)*
basal: 11 (6–17)	**2**	73	(33–227)*	86	(59–108)*	79	(46–112)*	63	(36–89)*
	**3**	NA		NA		43	(19–135)*	59	(23–169)*
	**4**	NA		NA		26	(6–61)	NA	
**TNFα** †	**1**	10	(0.1–17)	8	(1–22)*	11	(1–30)*	12	(0.4–17)*
basal: 0.4 (0.2–5)	**2**	15	(3–28)*	11	(0.4–32)*	15	(1–32)*	6	(0.4–21)*
	**3**	NA		NA		2	(0.3–35)	2	(2–23)
	**4**	NA		NA		4	(1–9)	NA	
**Anti-inflammatory cytokines**									
**IL-10** ††	**1**	824	(416–2126)*	991	(578–1724)*	2445	(1834–4053)*	2168	(1348–3753)*
basal: 273 (157–355)	**2**	792	(389–4168)*	1346	(568–1923)*	2016	(844–2741)*	876	(541–1837)*
	**3**	NA		NA		2923	(862–1234)*	1903	(862–3330)*
	**4**	NA		NA		3740	(1154–4051)*	NA	
**IL-13** ††	**1**	976	(678–2073)*	1481	(567–3274)*	2739	(571–3691)*	1628	(531–3958)*
basal: 215 (173–251)	**2**	1456	(565–4134)*	792	(275–1145)*	1368	(282–1765)*	527	(244–959)*
	**3**	NA		NA		262	(207–490)	327	(194–592)
	**4**	NA		NA		338	(123–511)	NA	

Mice were intratracheally infected with *A. baumannii* RUH875, RUH134, LUH5875 or LUH8326 for 1–4 days. Levels of inflammatory mediators were determined in the lung homogenates of mice directly after instillation (basal values), and 1–4 days after instillation. Results are medians and ranges for 8 mice, except for LUH8326 at 3 days after infection, where n = 4. Values are representative for surviving mice only. NA, not assessable, due to the high mortality associated with these strains.

*, significantly (p<0.05) different from basal level.

† , results in ng/g of lung tissue;

†† , results in pg/g of lung tissue.

In serum, all cyto- and chemokine levels were elevated 1 day after infection with RUH875, RUH134, LUH5875 and LUH8326, except for IL-23 that was not detectable (Table S1 in the online data supplement). For the majority of the cyto- and chemokines measured (KC, MIP-1α, MIP-2, IL-1β, IL-6 and TNFα), levels in the lungs corresponded to levels in serum. However, the kinetics of the innate response in lungs and serum differed, with some cyto- and chemokines peaking earlier (IL-1β and IL-10) and some later (MIP-1α, MIP-2, RANTES and TNFα) in serum than in the lungs of infected animals. No significant differences in levels of inflammatory mediators were seen between RUH875, RUH134, LUH5875 and LUH8326. Infection of mice with RUH3023^T^ and *A. junii* LUH5851 did not cause an increase in inflammatory mediators in serum, except for a 5-fold increase of RANTES and IL-1β levels (Table S1).

Overall, cyto- and chemokine levels in lungs and serum correlated (p<0.05) to bacterial levels in lungs and blood, respectively. We determined whether there was a correlation between cyto- and chemokine production at day 1 and tissue pathology, morbidity and mortality of mice at days 1–3 after infection with *A. baumannii* RUH875, RUH134, LUH5875, and LUH8326. Results revealed that low levels of IL-10 in lungs of mice were associated with severe lung pathology (r = −0.70, p<0.05) at the first day of infection. Morbidity was associated with low IL-10 levels (r = −0.72, p<0.001), low IL-12p40 (−0.37, p<0.05) and IL-23 (r = −0.67, p<0.001) levels in the lungs. Moreover, low levels of IL-10 in lungs at day 1 correlated to mortality at days 2 (r = −0.77; p<0.05) and 3 of infection (r = −0.72, p<0.01). A similar correlation was found between IL-12p40 and IL-23 levels at day 1 and mortality at days 2 (r = −0.37, p<0.05 and r = −0.71, p<0.01, respectively) and 3 (r = −0.35, p<0.05 and r = −0.67, p<0.01, respectively).

## Discussion

The outcome of pneumonia differed strikingly among *A. baumannii* strains with clone I and II and the sporadic isolate being highly virulent and the clone III strain (a clone less widespread than clones I and II) being less virulent. Infection of mice with type strain RUH3023^T^ and *A. junii* LUH5851 did not result in morbidity/mortality.

The clone I, II, III strains and the sporadic strain survived and multiplied in the lungs and disseminated to the bloodstream at high levels. Difference in mortality between clone III versus clone I, II and the sporadic strain could not be attributed to bacterial loads in lungs or blood, suggesting that proliferation in lungs and extrapulmonary dissemination are not the only factors contributing to the virulence of these strains.

We previously demonstrated *in vitro* that *A. junii* strains induced a stronger innate immune response in human cells than *A. baumannii* strains, implying that *A. junii* may be quickly eliminated by the host [5]. The finding that *A. junii* LUH5851 did not survive in our pneumonia model supports this presumption, although experiments focusing on the first hours of infection are necessary to assess the relationship between clearance and the innate immune response. Multiple factors play a role in clearance of pathogens from the lungs, including phagocytosis and killing by neutrophils and macrophages, by antimicrobial peptides and serum components [16]. As we used neutropenic mice, other factors than neutrophils contributed to the rapid clearance of the *A. junii* strain and RUH3023^T^. It is of note that RUH3023^T^ was more susceptible to killing by human serum *in vitro* than *A. baumannii* RUH875, RUH134, LUH5875 and LUH8326 (de Breij et al, unpublished).

The type strain of *A. baumannii* (ATCC19606^T^) was used as a model strain in several virulence studies [9], [17]–[21]. The finding that this strain, in contrast to other *A. baumannii* strains, did not survive in the mouse pneumonia model and in a mouse thigh infection model [22] (de Breij et al, unpublished), challenges the relevance of this strain as representative for the *A. baumannii* species. Altogether, noted differences in virulence among *A. baumannii* strains, as also observed by others [15], [23], underscore that the choice of strain is a critical variable in virulence studies. The reference strains of EU clones I and II that are associated with outbreaks worldwide were highly virulent in our study. It is important to further assess whether this is a general feature of these clones as this might have implications for clinical diagnostics [24], [25].

Eveillard et al [15] described a significant increase in TNFα and MIP-2 levels in lungs of mice after infection with five different *A. baumannii* strains. They showed that MIP-2 levels were higher in mice after the second day of infection with two virulent strains than with three less-virulent strains. Further to this, we found a clear association with the severity of infection and levels of the anti-inflammatory cytokine IL-10. The effects of IL-10 during bacterial infections are complex. During an overwhelming infection, as in our mouse studies, the anti-inflammatory effects of IL-10 are most likely beneficial to the host by down-regulating inflammation and its unfavourable effects [26], [27]. However, IL-10 also hampers the appropriate pro-inflammatory response to the bacteria, and then it can be hazardous for the host [28], [29]. Indeed, we also found that low levels of IL-12p40 and IL-23 were associated with a poor outcome, which is in agreement with Happel et al, who demonstrated the critical roles of IL-12p40 and IL-23 in host survival in a murine model of *Klebsiella pneumoniae* infection [30]. Others reported that increased levels of IL-12p40 as well as TNFα and IL-4 in neutropenic mice infected with *Cryptococcus neoformans* were associated with survival of these mice but not with a decreased fungal burden [31]. IL-23 is a cytokine together with enhanced IL-1β and IL-6 production known to drive an IL-17-producing T cell population in mice [32] that enhance epidermal defence and neutrophil influx. However, it is uncertain whether this cytokine plays a crucial role in host defence against *A. baumannii* as IL-17 depletion did not increase mortality in *A. baumannii* infected mice [23].

In conclusion, a striking difference in morbidity and mortality associated with *A. baumannii* strains was noted, with EU clone I and II strains being the most virulent. Furthermore, the outcome of experimental *A. baumannii* pneumonia is associated with IL-10 and IL-12p40/IL-23 levels. Future studies will have to clarify whether this response influences the impact of *A. baumannii* strains in the human host. If so, levels of these mediators may have predictive values or be targets for treatment.

## Materials and Methods

### Bacteria

Five *A. baumannii* strains, including reference strains of EU clones I–III, the type strain (RUH3023^T^ = ATCC19606^T^) and a sporadic isolate (LUH8326), and one *A. junii* strain were investigated (Table 1). Bacteria were preserved in glycerolbroth at −80°C. Prior to experiments, strains were rendered virulent by a single passage in mice.

### Animals

Specific pathogen-free female C3H/HeN mice weighing 18–20 g were housed fifteen per cage and had *ad libitum* access to chow and water throughout the experiments. Animal studies were approved by the Animal Experimental Committee of the Angers University Hospital (permit C49007002) and complied with relevant laws related to the conduct of animal experiments.

### Mouse pneumonia

The survival of *Acinetobacter* strains after intratracheal infection of mice was assessed according to Eveillard et al [15]. To favour the onset of infection, mice were rendered transiently neutropenic by intraperitoneal injection with 150 mg of cyclophosphamide per kg of body weight (in 100 µl of saline) at days 4 and 3 prior to infection. Bacteria from an overnight culture on blood agar were suspended into saline to an optical density of 0.5 McFarland, corresponding to a concentration of approximately 10^8^ colony forming units (CFU)/ml. Mice were anesthesized by isoflurane in conjunction with oxygen and 50 µl of the bacterial suspension were injected intratracheally via a cannula. Immediately after inoculation, two animals were sacrificed, lungs were homogenized and vital count was performed to verify the infection inoculum (range 5.9×10^5^–4.7×10^6^ CFU/g lung tissue). At 1, 2, 3 and 4 days after infection, if possible 8 mice per strain were anesthesized and blood was collected by intracardiac puncture, after which mice were sacrificed by cervical dislocation. Serum was obtained by centrifugation of blood samples and stored at −80°C for cytokine analysis. Spleens and part of the lungs were removed, weighed and homogenized in 3 ml of phosphate buffered saline (pH 7.4) using the GentleMACS Dissociator (Miltenyi Biotec, Germany). Vital counts in blood, lung and spleen homogenates were performed to assess the number of viable bacteria (lowest limit of detection: 20 CFU/ml). Lung homogenates were stored at −80°C for cytokine analysis.

A semi-quantitative analysis of mice morbidity was performed using a clinical score ranging from 0 for no clinical symptoms to 4 for maximal symptoms based on the following criteria: mice mobility (0, spontaneous; 1, only after stimulation; 2, absent), the development of conjunctivitis (0, absent; 1, present), and the aspect of the hair (0, normal; 1 ruffled). Mortality was assessed daily and analyzed by Kaplan-Meier survival curve.

### Histological analysis of lung inflammation

Lungs of mice at day 1–4 after inoculation were analyzed by histological examination as described [15]. A semi-quantitative analysis of the lung tissue damage was performed by grading five random 20× fields of hematoxylin/eosin-stained sections according to the following criteria: alveolar wall destruction [absent (0); <25% (1), 25–75% (2), >75% (3) of alveoli destructed], infiltration by leukocytes [absent (0); <20 (1), 20–50 (2), >50 (3) per field] and hemorrhage [absent (0); mild (1); moderate (2); severe(3)]. The sum of scores represents the lung pathology score ranging from 0 for no pathology to 9 for severe pathology.

### Determination of inflammatory mediators

Levels of interleukin (IL)-1β, IL-6, IL-10, IL-12p40, IL-13, IL-23, keratinocyte-derived chemokine (KC), macrophage inflammatory protein (MIP)-1α, MIP-2, regulated upon activation, normal T cell expressed and secreted (RANTES) and tumor necrosis factor (TNF)α in serum and lung homogenates were determined using multiplexing xMAP technology (Luminex Corporation Austin, USA). Multiplex kits were from Millipore (Millipore Corporation, USA).

### Statistical analysis

Data were analyzed using the Kruskal-Wallis one-way analysis of variance and Wilcoxon rank sum test (SPSS 17.0). Mortality data were analyzed by Cox-regression. Spearman rank correlation coefficients were calculated to evaluate associations between parameters. P≤0.05 were considered significant.

## Supporting Information

Table S1 **Inflammatory response in serum of mice infected with** ***Acinetobacter*** **.** Mice were intratracheally infected with *A. baumannii* RUH875, RUH134, LUH5875, LUH8326, RUH3023^T^ or *A. junii* LUH5851 for 1–4 days. Levels of inflammatory mediators were determined in the serum of mice directly after instillation (basal values), and 1–4 days after instillation. Results are median and ranges for 8 mice, except for LUH8326 at 3 days after infection, where n = 4. Values are representative for surviving mice only. NA, not assessable, due to the high mortality associated with these strains. *, significantly (p<0.05) different from basal level.
(DOC)
