# Supplementary material for: Differences in Acinetobacter baumannii Strains and Host Innate Immune Response Determine Morbidity and Mortality in Experimental Pneumonia
Source: PLoS One. 2012 Feb 8;7(2):e30673. doi: 10.1371/journal.pone.0030673 (PMC3275605; doi:10.1371/journal.pone.0030673)
Supplement: Table S1 — Inflammatory response in serum of mice infected with Acinetobacter . Mice were intratracheally infected with A. baumannii RUH875, RUH134, LUH5875, LUH8326, RUH3023T or A. junii LUH5851 for 1–4 days. Levels of inflammatory mediators were determined in the serum of mice directly after instillation (basal values), and 1–4 days after instillation. Results are median and ranges for 8 mice, except for LUH8326 at 3 days after infection, where n = 4. Values are representative for surviving mice only. NA, not assessable, due to the high mortality associated with these strains. *, significantly (p<0.05) different from basal level. (DOC) [file pone.0030673.s001.doc]

**Differences in *Acinetobacter baumannii* Strains and Host Innate Immune Response Determine Morbidity and Mortality in Experimental Pneumonia**

Anna de Breij, Matthieu Eveillard, Lenie Dijkshoorn, Peterhans J. van den Broek, Peter H. Nibbering, Marie-Laure Joly-Guillou

**Online data supplement**

***Table S1. Inflammatory response in serum of mice infected with Acinetobacter***

|  |  | **RUH875** | |  | **RUH134** | |  | **LUH5875** | |  | **LUH8326** | |  | **RUH3023T** | |  | **LUH5851** | |
| --- | --- | --- | --- | --- | --- | --- | --- | --- | --- | --- | --- | --- | --- | --- | --- | --- | --- | --- |
|  |  |  |  |  |  |  |  |  |  |  |  |  |  |  |  |  |  |  |
| **Chemokines** | **Day** |  |  |  |  |  |  |  |  |  |  |  |  |  |  |  |  |  |
|  |  |  |  |  |  |  |  |  |  |  |  |  |  |  |  |  |  |  |
| **KC** | **1** | 60 | (7 - 256) * |  | 57 | (7 - 251) * |  | 46 | (1 - 346) * |  | 67 | (7 - 145) * |  | 1 | (0.2 - 4) |  | 8 | (0.2 - 11) * |
| basal: 0.1 (0.03 - 0.6) | **2** | 21 | (4 - 54) * |  | 9 | (2 - 674) * |  | 12 | (2 - 80) * |  | 6 | (0.2 - 72) * |  | 0.2 | (0.1 - 0.4) |  | 0.4 | (0.2 - 5) |
|  | **3** | NA |  |  | NA |  |  | 0.1 | (0 - 93) |  | 0.01 | (0 - 0.02) |  | 0 | (0 - 0.03) |  | 0 | (0 - 0.2) |
|  | **4** | NA |  |  | NA |  |  | 0.1 | (0 - 1) |  | NA d |  |  | 0 |  |  | 0 |  |
|  |  |  |  |  |  |  |  |  |  |  |  |  |  |  |  |  |  |  |
| **MIP-1α** | **1** | 0.4 | (0.1 - 2) * |  | 0.3 | (0.1 - 1) * |  | 0.2 | (0.1 - 0.3) * |  | 0.1 | (0.1 - 0.3) * |  | 0.1 |  |  | 0.1 |  |
| basal: 0.1 | **2** | 2 | (1 - 11) * |  | 2 | (0.1 - 5) * |  | 4 | (0.1 - 17) * |  | 1 | (0.1 - 4) * |  | 0.1 |  |  | 0.1 |  |
|  | **3** | NA |  |  | NA |  |  | 7 | (0.1 - 84) * |  | 2 | (0.4 - 82) * |  | 0.1 |  |  | 0.1 |  |
|  | **4** | NA |  |  | NA |  |  | 6 | (0.1 - 23) * |  | NA nd |  |  | 0.1 |  |  | 0.1 |  |
|  |  |  |  |  |  |  |  |  |  |  |  |  |  |  |  |  |  |  |
| **MIP-2** | **1** | 18 | (1 - 25) * |  | 11 | (2 - 23) * |  | 5 | (0.1 - 13) * |  | 11 | (2 - 19) * |  | 0.2 | (0.1 - 0.2) |  | 0.2 | (0.1 - 1) |
| basal: 0.1 (0.1 - 0.2) | **2** | 10 | (1 - 22) * |  | 8 | (0.1 - 18) * |  | 10 | (0.3 - 14) * |  | 3 | (0.1 - 10) |  | 0.1 |  |  | 0.1 |  |
|  | **3** | NA |  |  | NA |  |  | 0.5 | (0.1 - 65) |  | 0.1 | (0.1 - 58) |  | 0.1 |  |  | 0.1 | (0.1 - 0.2) |
|  | **4** | NA |  |  | NA |  |  | 0.1 | (0.1 - 1) |  | NA nd |  |  | 0.1 |  |  | 0.1 |  |
|  |  |  |  |  |  |  |  |  |  |  |  |  |  |  |  |  |  |  |
| **RANTES** | **1** | 49 | (4 - 127) * |  | 31 | (12 - 101) * |  | 19 | (4 - 53) * |  | 23 | (4 - 50) * |  | 18 | (12 - 32) * |  | 25 | (9 - 46) * |
| basal: 4 (4 - 6) | **2** | 27 | (4 - 59) |  | 21 | (4 - 126) * |  | 22 | (4 - 50) * |  | 16 | (4 - 38) |  | 13 | (12 - 21) * |  | 15 | (12 - 19) |
|  | **3** | NA |  |  | NA |  |  | 10 | (4 - 113) |  | 6 | (4 - 110) |  | 9 | (4 - 21) |  | 8 | (4 - 32) * |
|  | **4** | NA |  |  | NA |  |  | 15 | (12 - 30) * |  | NA nd |  |  | 11 | (9 - 18) * |  | 12 | (9 - 21) * |
|  |  |  |  |  |  |  |  |  |  |  |  |  |  |  |  |  |  |  |
| **Pro-inflammatory cytokines** | |  |  |  |  |  |  |  |  |  |  |  |  |  |  |  |  |  |
|  |  |  |  |  |  |  |  |  |  |  |  |  |  |  |  |  |  |  |
| **IL-1β** | **1** | 393 | (5 - 845) * |  | 185 | (102 - 550) * | | 234 | (5 - 617) * |  | 225 | (55 - 1002) * | | 119 | (119 - 223) * |  | 62 | (5 - 119) |
| basal: 30 (5 - 55) | **2** | 144 | (55 - 617) * |  | 144 | (5 - 584) |  | 411 | (55 - 684) * |  | 78 | (55 - 908) * |  | 62 | (5 - 119) |  | 119 | (119 - 223) * |
|  | **3** | NA |  |  | NA |  |  | 78 | (5 - 1002) |  | 30 | (5 - 1495) |  | 119 | (5 - 119) |  | 119 | (5 - 119) * |
|  | **4** | NA |  |  | NA |  |  | 119 | (56 - 223) * |  | NA nd |  |  | 119 | (5 - 223) |  | 119 | (5 - 223) * |
|  |  |  |  |  |  |  |  |  |  |  |  |  |  |  |  |  |  |  |
| **IL-6** | **1** | 33 | (2 - 99) * |  | 27 | (7 - 95) * |  | 30 | (1 - 84) * |  | 40 | (1 - 105) * |  | 0 | (0 - 1) |  | 5 | (0 - 10) |
| basal: 0.01 (0.01 - 0.1) | **2** | 27 | (9 - 79) * |  | 16 | (0.3 - 633) * |  | 34 | (5 - 181) * |  | 8 | (0 - 158) * |  | 0 |  |  | 0 | (0 - 1) |
|  | **3** | NA |  |  | NA |  |  | 1 | (0 - 151) * |  | 1 | (0.1 - 134) * |  | 0 |  |  | 0 | (0 - 0.1) |
|  | **4** | NA |  |  | NA |  |  | 0.2 | (0 - 4) |  | NA nd |  |  | 0 |  |  | 0 |  |
|  |  |  |  |  |  |  |  |  |  |  |  |  |  |  |  |  |  |  |
| **IL-12p40** | **1** | 42 | (20 - 173) * |  | 27 | (20 - 85) |  | 25 | (20 - 75) |  | 20 | (20 - 28) |  | 20 |  |  | 20 | (20 - 22) |
| basal: 20 | **2** | 20 | (20 - 61) |  | 20 | (20 - 72) |  | 20 | (20 - 50) |  | 20 | (20 - 57) |  | 20 |  |  | 20 |  |
|  | **3** | NA |  |  | NA |  |  | 20 | (20 - 85) |  | 20 |  |  | 20 |  |  | 20 |  |
|  | **4** | NA |  |  | NA |  |  | 20 | (20 - 27) |  | NA nd |  |  | 20 |  |  | 20 |  |
|  |  |  |  |  |  |  |  |  |  |  |  |  |  |  |  |  |  |  |
| **TNFα** | **1** | 128 | (3 - 285) * |  | 84 | (5 - 223) * |  | 70 | (3 - 97) * |  | 55 | (3 - 110) * |  | 3 |  |  | 24 | (3 - 24) * |
| basal: 3 | **2** | 259 | (84 - 345) * |  | 191 | (3 - 446) * |  | 185 | (3 - 450) * |  | 77 | (3 - 540) * |  | 3 | (3 - 24) |  | 3 | (3 - 24) |
|  | **3** | NA |  |  | NA |  |  | 121 | (3 - 3749) * |  | 21 | (3 - 2295) |  | 3 | (3 - 24) |  | 3 | (3 - 24) |
|  | **4** | NA |  |  | NA |  |  | 24 | (3 - 234) * |  | NA nd |  |  | 3 | (3 - 82) |  | 3 | (3 - 24) |
|  |  |  |  |  |  |  |  |  |  |  |  |  |  |  |  |  |  |  |
| **Anti-inflammatory cytokines** | |  |  |  |  |  |  |  |  |  |  |  |  |  |  |  |  |  |
|  |  |  |  |  |  |  |  |  |  |  |  |  |  |  |  |  |  |  |
| **IL-10** | **1** | 992 | (45 - 1271) * | | 373 | (16 - 2127) * | | 45 | (16 - 1023) * | | 159 | (45 - 528) * |  | 20 | (16 - 124) * |  | 20 | (20 - 92) * |
| basal: 16 | **2** | 620 | (237 - 1299) * | | 147 | (35 - 519) * |  | 319 | (16 - 1528) * | | 159 | (16 - 552) * |  | 18 | (16 - 20) |  | 20 | (20 - 75) * |
|  | **3** | NA |  |  | NA |  |  | 390 | (16 - 45053) * | | 305 | (16 - 1541) * | | 20 | * |  | 20 | (16 - 58) |
|  | **4** | NA |  |  | NA |  |  | 1475 | (20 - 3020) * | | NA nd |  |  | 30 | (20 - 92) * |  | 20 | (16 - 58) * |
|  |  |  |  |  |  |  |  |  |  |  |  |  |  |  |  |  |  |  |
| **IL-13** | **1** | 2 | (1 - 3) * |  | 2 | (1 - 2) |  | 2 | (1 - 2) |  | 2 | (1 - 3) * |  | 2 | (1 - 2) |  | 1 | (1 - 2) |
| basal: 1 (1 - 2) | **2** | 1 | (1 - 2) |  | 2 | (1 - 2) |  | 2 | (1 - 4) |  | 2 | (1 - 2) * |  | 2 | (1 - 2) |  | 1 | (1 - 2) |
|  | **3** | NA |  |  | NA |  |  | 1 | (1 - 2) |  | 1 | (1 - 2) |  | 1 | (1 - 2) |  | 1 | (1 - 2) |
|  | **4** | NA |  |  | NA |  |  | 1 | (1 - 2) |  | NA nd |  |  | 1 | (1 - 2) |  | 1 | (1 - 2) |
|  |  |  |  |  |  |  |  |  |  |  |  |  |  |  |  |  |  |  |

Mice were intratracheally infected with *A. baumannii* RUH875, RUH134, LUH5875, LUH8326, RUH3023^T^ or *A. junii* LUH5851 for 1-4 days. Levels of inflammatory mediators were determined in the serum of mice directly after instillation (basal values), and 1-4 days after instillation. Results are median and ranges for 8 mice, except for LUH8326 at 3 days after infection, where n=4. Values are representative for surviving mice only. NA, not assessable, due to the high mortality associated with these strains. *, significantly (p < 0.05) different from basal level.
